# Structural Variability of Pfam Domains Based on Alphafold2 Predictions

**DOI:** 10.1002/prot.70021

**Published:** 2025-07-22

**Authors:** Elly Poretsky, Carson M. Andorf, Taner Z. Sen

**Affiliations:** ^1^ Crop Improvement and Genetics Research Unit, United States Department of Agriculture, Agricultural Research Service Western Regional Research Center Albany California USA; ^2^ Corn Insects and Crop Genetics Research Unit, United States Department of Agriculture Agricultural Research Service Ames Iowa USA; ^3^ Department of Computer Science Iowa State University Ames Iowa USA; ^4^ Department of Bioengineering University of California Berkeley California USA

**Keywords:** AlphaFold2, Pfam, protein functional prediction, protein structure prediction, protein tertiary structure prediction

## Abstract

Understanding the biological functions of proteins is one of the main goals of functional genomics. Such understanding will help control and manipulate biological processes to enhance desirable traits, including improved abiotic and biotic stress resistance in humans, animals, plants, and microbes. Protein domains, regarded as the functional building blocks of proteins, have been used extensively to predict protein function. Sequence‐based approaches for protein function prediction, including the use of protein domain prediction from resources like the Pfam database, remain popular due to their reliability, low cost, and ease of use. Although the sequence variability of Pfam domains has been reported in several studies, their structural variability has been understudied. Here, we have extracted the Pfam domain structural portion from the predicted structures of the 16 model organism proteomes in the AlphaFold2 database. Our analysis revealed that many families contained between 20% and 40% members with no assigned regular secondary structures, demonstrating within‐family structural variability. To better understand this structural variability, we used FoldSeek and agglomerative clustering to identify structural variability in Pfam families. We then analyzed specific cases to provide structural details for this variability. In this study, we have used two popular prediction applications/resources, Alphafold2 and Pfam, to demonstrate inherent variability in protein domain predictions by comparing their predicted structures. Our study shows that detection of structural variability in Pfam families can facilitate curation and refinement of Pfam families, while demonstrating the need to develop more accurate protein domain prediction workflows.

**Abbreviations**
ARS: agricultural research service
CASP: critical assessment of structure prediction
DOE: Department of Energy
EBI: European Bioinformatics Institute
HMM: Hidden Markov Model
ORISE: Oak Ridge Institute for Science and Education
PCA: principal component analysis
PDB: protein data bank
Pfam: protein families database
pLDDT: predicted local distance difference test
USDA: the U.S. Department of Agriculture

## 1 Introduction

Protein domains are viewed as the functional building blocks that carry specific and conserved functions [1]. For this reason, the annotation and prediction of protein domains in large databases have been instrumental for comparative analyses and predicting protein functions [2]. Due to the ubiquity of protein sequence data, protein domain annotations have been largely reliant on sequence‐based annotations, such as the Pfam domain prediction method currently implemented in the InterPro database [3, 4]. Nevertheless, the growing number of solved structures in the PDB database has been used to develop protein domain databases that relied, in part, on information derived from sequence‐based protein domain annotations [5, 6]. Furthermore, recent developments in artificial intelligence‐based methods for highly accurate prediction of protein structures enabled the development of large‐scale protein structure databases [7, 8]. While sequence‐based domain predictions remain popular for protein function annotations, recent developments in protein structure predictions facilitated protein domain discoveries on the structural levels for databases such as CATH and The Encyclopedia of Domains [9].

After plateauing for several years [10], the success in protein tertiary structure prediction leaped first in 2018 and then again in 2020 by the introduction of AlphaFold and AlphaFold2 [7] by DeepMind. At the biennially organized Critical Assessment of Structure Prediction (CASP) community contest, the last of which (CASP15) was held in 2022 [11], the global distance test parameter is used to assess predicted structures against experimentally determined structures based on amino acid residue positions. Until 2016, this parameter was around 40 (out of 100). Then, in 2018 and 2020, AlphaFold first surpassed the 50 threshold and then 80, respectively, demonstrating that it can predict protein structures almost at the same level as determined by experiments. AlphaFold does not always provide the most accurate structures and certainly has limitations [12], but it is also true that AlphaFold is currently the most powerful protein structure predictor, especially given the fact that RoseTTAFold2 [13], which was the best‐performing group at CASP15 that did not use AlphaFold, ranked 28th after those who did [11].

While protein structures are often viewed as static entities, proteins often exist as ensembles of conformations, and a better understanding of the structural dynamics is important for understanding protein function [14, 15, 16]. When considering the structural variability of proteins in nature and in prediction methods, different factors may be considered [7, 17]. For instance, intrinsic factors, such as sequence variation, post‐translational modifications, physical dynamics, conformational flexibility, and disordered regions can alter protein folding and increase the observed structural variability [16, 18, 19, 20]. Extrinsic factors, such as hydrophobicity, pH, protein–protein interactions, and ligand binding can influence structural conformation and folding pathways that are not captured by single predicted structures [21, 22, 23]. These biochemical and physiochemical factors have a direct impact on the captured structural diversity available for training structure prediction methods such as AlphaFold2. Thus, disproportionately well‐characterized protein families and gaps in captured protein conformers can introduce biases that reduce protein structure prediction accuracy, limit predicted structural diversity, or generate unrealistic structures [24, 25]. Additionally, the depth and quality of multiple sequence alignments significantly affect the prediction accuracy of protein structures to the extent that it can itself be modified to capture a broader range of biologically relevant conformational states [7, 26]. Together, these factors highlight the complexity of protein structure prediction and underscore the importance of understanding structural diversity in informing protein function.

InterPro is one of the most widely used tools for protein function annotation. By integrating a large number of annotation methods, including the Protein families (Pfam) protein domain annotation database, InterPro provides comprehensive information that can be used to elucidate gene functions. Large‐scale comparative analyses have been previously applied to protein sequences and structures to identify and annotate new protein families and domains. For example, using sequence similarity to analyze UniProt proteins with high‐confidence AlphaFold models has facilitated the discovery of new structural folds and new Pfam families [27], with the aim of identifying, prioritizing, and annotating novel protein families, superfamilies, and folds. Inter‐domain analysis compares structures across different Pfam domains to explore evolutionary relationships and structural convergence/divergence [28]. By leveraging established databases together with the recent advancements in protein structure predictions, we can shed light into uncharted areas of the protein universe at an unprecedented scale, paving the way to innovations in life sciences and biotechnology. In this work, we focus on AlphaFold2‐predicted structures of Pfam domains to analyze the structural diversity of Pfam domains and provide evidence for the possible implications for protein structural variation that may lead to inaccurate functional predictions.

## 2 Methods

### 2.1 Generation of Input Data

All the predicted AlphaFold2 protein sequences for the 16 representative model organisms with evolutionary diversity were downloaded from the EBI database AlphaFold Protein Structure Database (v2) [7, 8]. A total of 208 proteins with multiple AlphaFold2 predicted structures were removed from the analysis (Table S1). Pfam domains were predicted using InterProScan (v100) using the protein sequences extracted from the obtained PDB files (Table S2) ([3, 29]). We then used PDB‐tools (v2.0.0) to extract PDB files containing the residues associated with individual Pfam domains from the AlphaFold2 structures. After extracting the Pfam domain structures, BioPython (v1.85) was used to extract the Pfam domain protein sequences and pLDDT scores of the individual PDB files [30]. Additionally, STRIDE (v0.9.9) [31] was used to predict per‐residue secondary structures from the complete protein structures. Individual Pfam domains were then assigned into four broad categories: (1) α‐helix, if more than 20% of residues were predicted as ‘H’, ‘G’, or ‘I’, (2) β‐sheets, if more than 20% of residues were predicted as ‘E’ or ‘B’, (3) α‐helix and β‐sheet, if both categories were assigned, and (4) coils if neither categories were assigned. GNU parallel was used to run the workflow in parallel (v. 20231122).

### 2.2 Pfam Domain Structure Clustering and Merging

The extracted Pfam domain structure files, each Pfam domain separately, were clustered using FoldSeek (v10.941cd33) [32]. The clustering was conducted using the FoldSeek easy‐cluster function (–single‐step‐clustering 1, ‐c 0.9), which assigned all Pfam domain structures to individual clusters, designated by one cluster representative. Next, the FoldSeek easy‐search function was used to calculate the TM‐scores between all Pfam domain structures and the structures of the cluster representatives (Table S3) (–alignment‐type 1, –tmscore‐threshold 0.0, ‐format‐output query,target,alntmscore,u,t, –exhaustive‐search 1, ‐e inf) [33]. To reduce the number of clusters and to select for the clusters with the highest TM‐score differences, we used agglomerative clustering followed by merging of cluster representatives, selecting the representative with the highest average pLDDT score. Specifically, the Scikit‐learn (v1.6.1) AgglomerativeClustering function was used with the distance threshold set to represent TM‐score > 0.6 (linkage = ‘single’, metric = ‘precomputed’, distance_threshold = 0.4) (Table S3) [34].

### 2.3 Generation of Neighbor‐Joining Tree From Pfam Domain Sequences

The protein sequences of the Pfam domain structures were used to construct a maximum‐likelihood tree. First, the sequences were aligned using FAMSA (2.2.3‐5efa514; default parameters) [35], after which the alignment file was trimmed using ClipKIT (v2.3.0; default parameters) [36]. The ninja package (v2.1.11) [37] was used to infer the neighbor‐joining tree that was visualized using FigTree (v1.4.4; https://github.com/rambaut/figtree/releases).

## 3 Results and Discussion

### 3.1 Evidence for Structural Variability Across Pfam Families

**FIGURE 1:**
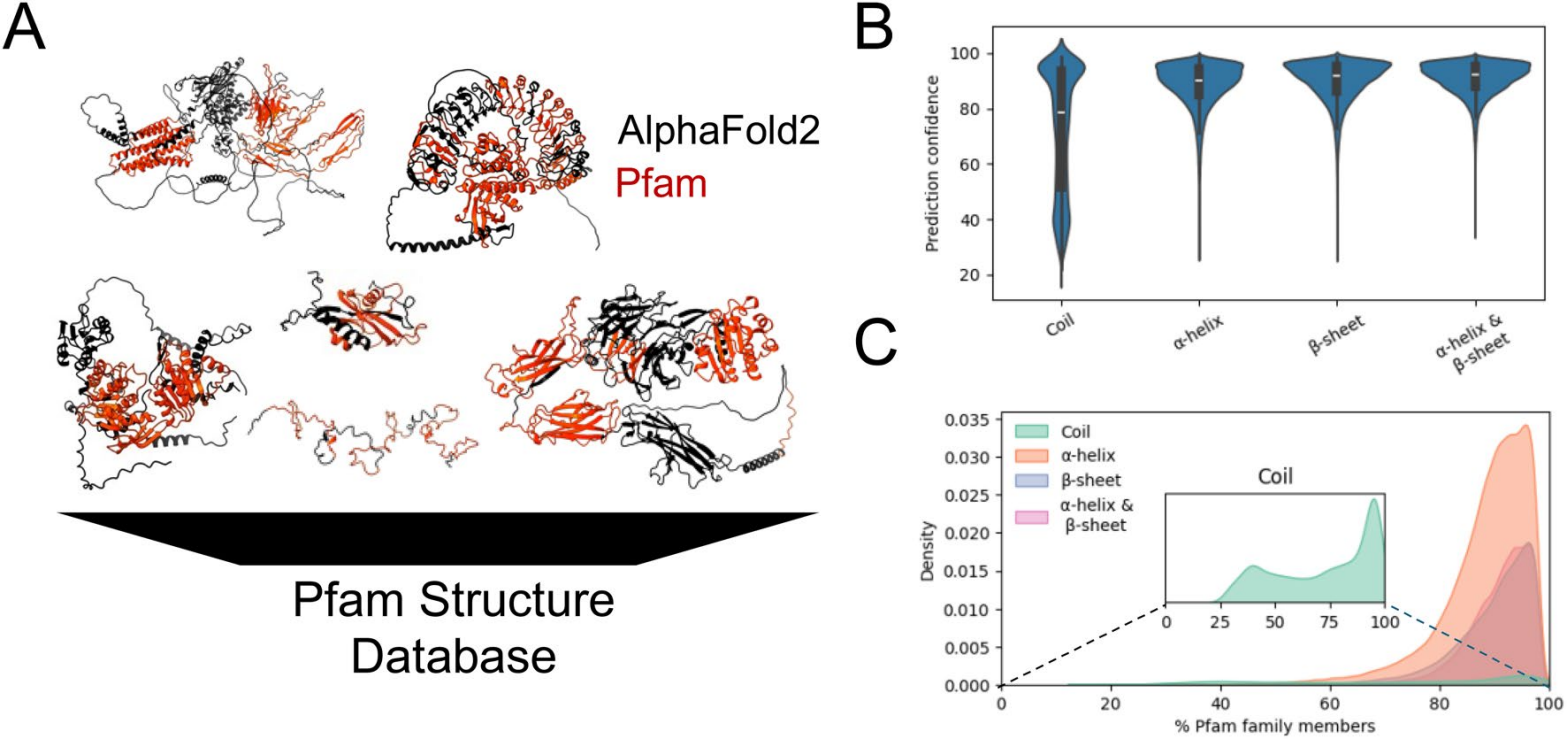
Structural variability among Pfam domain families. (A) InterProScan was used to predict Pfam domains using the protein sequences obtained from the 16 model organisms in the AlphaFold2 database. The boundaries of predicted Pfam domains were then used to extract the Pfam domain structures that were then assembled to generate a Pfam structure database for downstream analyses. (B) Distribution of prediction confidence scores, quantified as average pLDDT, of Pfam domain structures across four assigned secondary structure categories. (C) Distribution of the percentage of Pfam family members with assigned secondary structure categories aggregated over all Pfam families. An inset distribution plot was used to highlight the distribution of the coil secondary structure group independently from the regular secondary structure categories. Per‐residue secondary structures of complete protein structures were assigned using STRIDE. Individual Pfam domains were labeled α‐helices or β‐sheets if more than 20% of residues were assigned that category, α‐helix and β‐sheet if both categories were assigned, and coils if neither category were assigned.

As one of the most widely used tools for protein domain annotations, we used InterProScan to obtain the Pfam domain annotations for all available protein sequences in the AlphaFold2 predicted protein structures of the 16 reference genomes (Figure 1A, Table S2). We used the detected Pfam domain boundaries to extract the portions of the structure domains and their associated sequences for further comparative analysis (Figure 1A). To better understand the structural variability of Pfam families, we mapped the predicted Pfam domain boundaries to their predicted structures for the 16 model organisms in the AlphaFold2 database (Figure 1A) [8].

To understand the prediction confidence levels of AlphaFold structures, we used the predicted Local Distance Difference Test (pLDDT) scores. Figure 1B shows pLDDT scores for proteins with distinct secondary structure assignments, using a 20% threshold of domain residues to assign α‐helices and β‐sheets secondary structure category. Given that the pLDDT score is a measure of how well a given predicted structure would agree with its associated experimental structures, it is expected for regular secondary structures, that is, α‐helices and β‐sheets, to have a higher score, as illustrated in Figure 1B. On the other hand, Pfam domains assigned to the coil secondary structure category exhibited a bimodal distribution, where low pLDDT‐scoring domains likely correspond to relatively disordered regions, while high pLDDT‐scoring domains likely represent regions that adopt well‐defined but non‐canonical structures, such as loops or turns, which lack extensive α‐helices or β‐sheets but still exhibit stable and ordered conformations (Figure 1B).

We then tried to capture the distribution representing the percentage of Pfam family members assigned to each of the four secondary structure categories (Figure 1C). Note that due to the relatively low number of Pfam domains assigned to the coil secondary structure category, with previous work showing Pfam domains are biased toward structural order [38], an inset plot within the main plot was used to separately visualize the distribution of the coil secondary structure category (Figure 1C). Here, given that Pfam domains are predicted based on sequence conservation, the hypothesis was that members of the same Pfam families will have a relatively similar secondary structure composition. Under this hypothesis, we anticipated that the distribution of the percentage of Pfam family members assigned to each of the four secondary structure categories will peak close to 100% of the Pfam family members. Our analyses show that while the peak of the distributions of all four secondary structure categories is close to 100%, the left‐skewed distribution of the regular secondary structures and the bimodal distribution for the coil secondary structure show that many Pfam families contain a mix of members assigned to more than one secondary structure category (Figure 1C).

### 3.2 Agglomerative Clustering of FoldSeek Cluster Representatives Facilitates Merging of Structurally Similar Clusters

**FIGURE 2:**
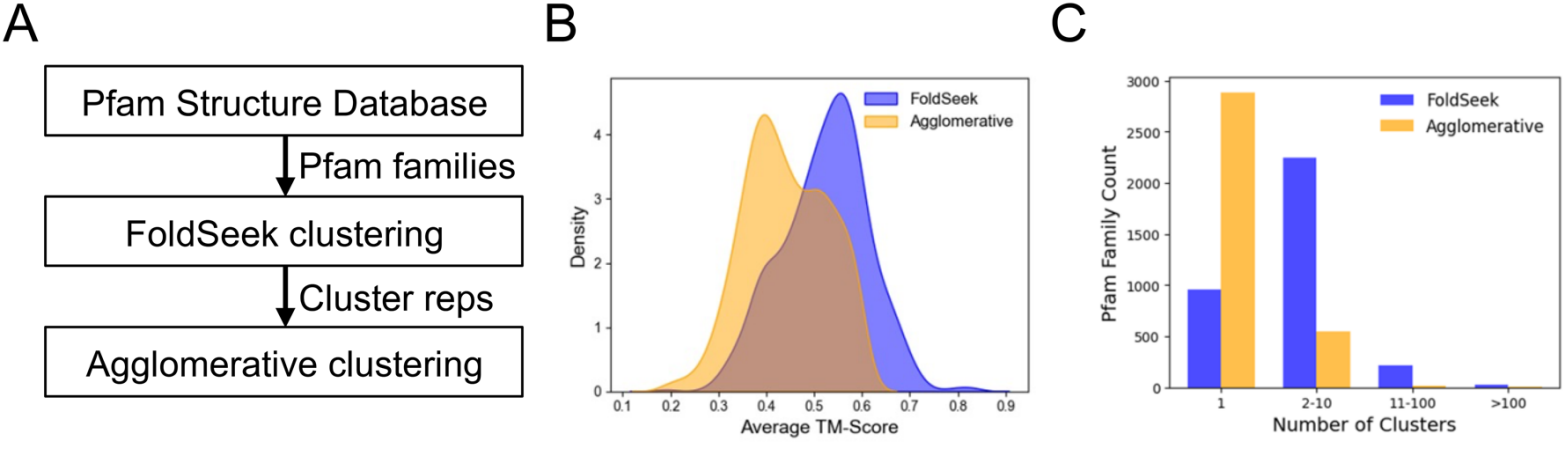
Two‐step clustering of individual Pfam families facilitates merging of structurally similar clusters. (A) The analysis workflow uses FoldSeek for an initial clustering of individual Pfam family structures, followed by agglomerative clustering of the FoldSeek cluster representatives. Results of the agglomerative clustering are then used to merge structurally similar FoldSeek clusters using a TM‐score threshold of 0.6. When merging clusters, the cluster with the higher average pLDDT score is chosen as new agglomerative cluster representative. (B) The distribution of within‐cluster average TM‐scores of FoldSeek cluster representatives and agglomerative cluster representatives, for each individual Pfam family with more than two agglomerative clusters. (C) The number of FoldSeek clusters and agglomerative clusters for each individual Pfam family.

The ability to detect Pfam domains using information from protein sequence alignments and Hidden Markov Model (HMM) profiles implies that domains belonging to the same Pfam families should also share structural similarities [29, 39]. Recently, FoldSeek developed an accurate and scalable method for clustering protein structures at the scale of the known protein universe [32]. Because FoldSeek clustering was developed to cluster whole protein structures and not portions of protein structures corresponding to individual domains, we considered using an additional clustering step that would facilitate merging of structurally similar FoldSeek cluster representatives. Thus, the Pfam domain structures of all individual Pfam families were initially clustered using FoldSeek, followed by agglomerative clustering of the FoldSeek cluster representatives (Figure 2A). Agglomerative clustering is a bottom‐up clustering approach that uses hierarchical clustering to iteratively merge clusters according to their similarity [40]. Here, agglomerative clustering was applied to the calculated TM‐scores between all FoldSeek cluster representatives and was used to effectively merge FoldSeek cluster representatives with a TM‐score threshold above 0.6 (Figure 2A). Focusing on the Pfam families that had more than one cluster remaining following the merging of agglomerative clusters (Table S3), we first analyzed the distribution of within‐cluster TM‐scores. As expected, the distribution of within‐cluster TM‐scores shifted to lower TM‐scores, from a median of 0.53 for the FoldSeek cluster representatives to 0.43 for the agglomerative clustering cluster representatives (Figure 2B). When comparing the number of FoldSeek cluster representatives before and after applying agglomerative clustering, we observed notable changes across different cluster‐size categories. The number of Pfam families represented by a single cluster significantly increased from 907 to 2837. Conversely, we saw a reduction in the number of Pfam families spanning “2 to 10” clusters, decreasing from 2251 to 547. Similarly, Pfam families with “11 to 100” clusters decreased from 220 to 16, and those containing “over 100 clusters” dropped from 26 to just 4 (Figure 2C). Thus, from an initial set of 11,785 Pfam families (Table S2), 3404 families with more than 20 members were clustered using FoldSeek to produce a set of 2497 Pfam families with two or more clusters (Tables S2 and S3), that were further clustered using agglomerative clustering to produce a final working set of 567 Pfam families with one or more clusters (Table S3). These results demonstrate that merging FoldSeek clusters through agglomerative clustering effectively consolidates structurally similar domains, highlighting the most structurally distinct Pfam domain clusters.

### 3.3 Structural Outliers Across Pfam Families Are Associated With Lower Prediction Confidence

**FIGURE 3:**
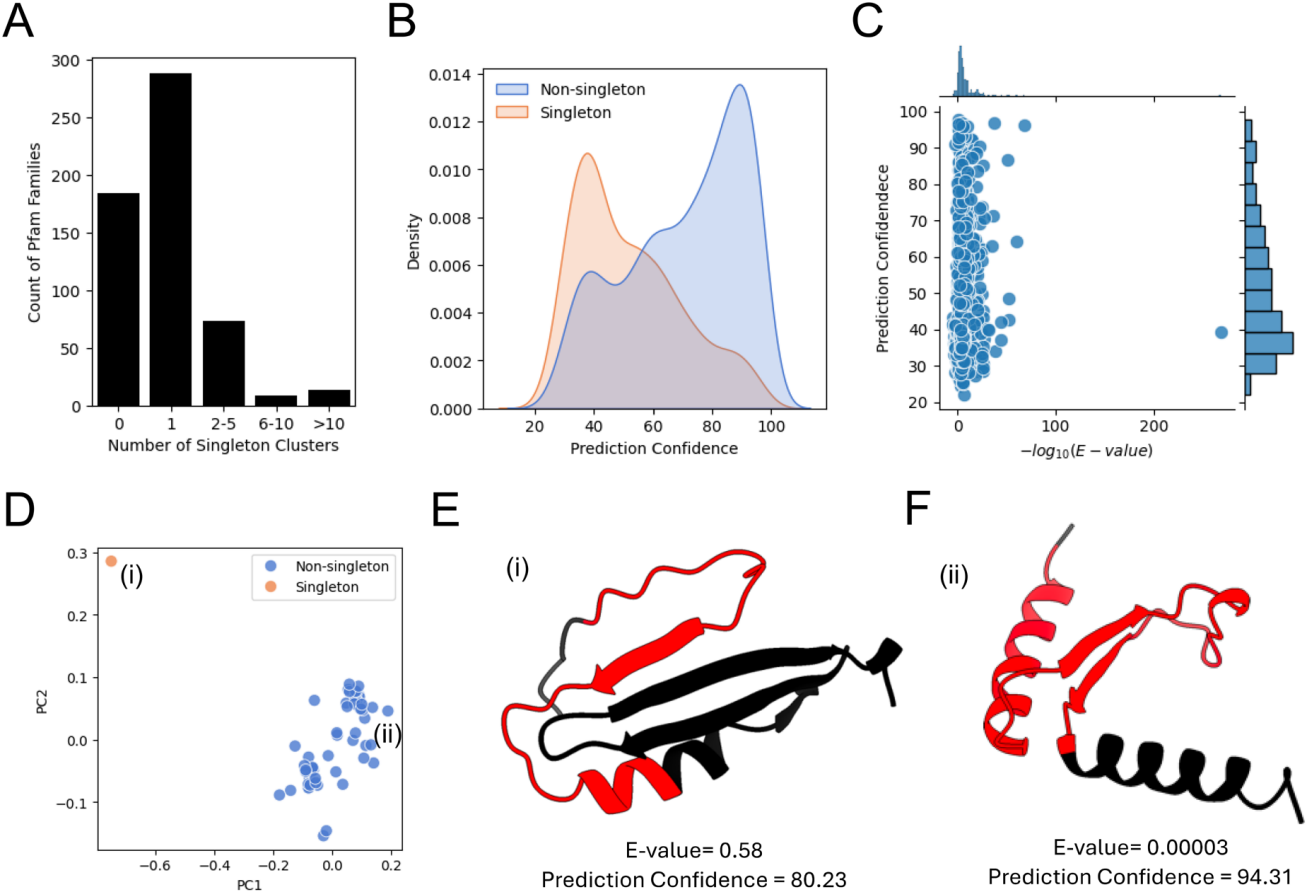
Clustering of Pfam domain structures facilitates the identification and annotation of structural outliers. (A) The number of Pfam families containing singleton clusters across different bins. (B) Distribution of structure prediction confidence, quantified as average pLDDT, of singleton and non‐singleton clusters representatives across the different Pfam families, excluding four Pfam families with more than 1000 singleton clusters. (C) Joint plot showing the distributions of –log_10_(*E*‐value) versus structure prediction confidence for singleton for the top singleton Pfam domain across Pfam families, excluding four Pfam families with more than 1000 singleton clusters. (D) PCA plot generated from the TM‐scores between the histidine triad motif (HIT) PF01230 Pfam family members with the color of individual domains representing non‐singleton and singleton cluster members referenced with roman numerals (i for A0A0N7KDY2 and ii for A0A0N7KQE7). Structure of (E) A0A0N7KDY2 (i) and (F) A0A0N7KQE7 (ii) with the PF13885 Pfam domains highlighted in orange across the full structures. Results include only Pfam families with more than two agglomerative clusters.

One of the outcomes of agglomerative merging is the ability to detect structural outliers based on the used TM‐score threshold. Focusing on Pfam families with a minimum of two clusters following the agglomerative clustering, we sought to identify Pfam domain structures that can be considered as structural outliers to demonstrate structural variability within families. Considering Pfam families which contained at least one singleton cluster, a cluster consisting of only one member, we found that of the 567 Pfam families included in the analysis, only 184 had no singleton clusters, 288 had one singleton cluster, 73 had between 2 and 10 singleton clusters, 9 had between 6 and 10 singleton clusters, and 13 had more than 10 singleton clusters (Figure 3A). We hypothesized that as possible structural outliers, singleton clusters might also have lower AlphaFold2 prediction confidence scores. By considering the distribution of the prediction confidence scores, quantified as the average pLDDT scores for the individual Pfam domain structures, we plotted the distributions of singleton and non‐singleton cluster representatives, showing that singleton clusters have substantially lower prediction confidence scores compared to non‐singleton clusters, with a median of 48.2 and 74.8, respectively (Figure 3B). Note that in order not to skew results, four Pfam families that contained more than 1000 singleton clusters were excluded from the analysis (Table S4). We found that each of these excluded Pfam families contained more singleton clusters than the rest of the Pfam families included in the analysis (Figure S1A), and that if we were to include them, the distribution of prediction confidence scores would have been highly skewed, with the median of the distribution increasing from 74.8 to 93.9, respectively (Figure S1B).

One of the possible reasons for the occurrence of singleton cluster structural outliers is due to false positive Pfam domain predictions, possibly showing low domain prediction confidence but high structure prediction confidence scores. To better understand the relationship between structure and domain prediction confidences, we plotted the structure prediction confidence scores, quantified as the average pLDDT, and compared it to the Pfam prediction confidence scores, quantified as the −log_10_(*e*‐value) reported by InterProScan, for the singleton Pfam domains. With both variables showing a skewed distribution toward lower confidence scores and no apparent clustering, the results suggest that there is no strong relationship between the two variables (Figure 3C). To look for specific examples, we identified the histidine triad motif (HIT) PF01230 Pfam family that has a singleton cluster with a low Pfam domain prediction score of 0.58 but a high structure prediction confidence score of over 80.23 (Figure 3D,E, Table S5). A PCA plot generated from the TM‐scores between the two cluster representatives and all the Pfam family members shows the singleton and non‐singleton cluster members, with the non‐singleton and singleton cluster representatives labeled with roman numerals (i for A0A0N7KDY2 and ii for A0A0N7KQE7, respectively) (Figure 3D). The structures of (i) A0A0N7KDY2 with a Pfam *e*‐value prediction score of 0.58 and structure prediction confidence scores of 80.23 and (ii) A0A0N7KQE7 with a Pfam *e*‐value prediction score of 0.00003 and structure prediction confidence scores of 94.31, with the PF13885 Pfam domains highlighted in orange showing the different secondary structure composition of the full structures and the PF13885 domains (Figure 3E,F, respectively). We then aligned the full protein sequences [41] and aligned the predicted protein structures [42] of the two representative clusters. The protein sequence alignment showed that the conserved HIT sequence motif HXHXHXX appears in the non‐singleton cluster, but not in the singleton cluster (Figure S3A) and the predicted protein structure alignment showed the two structures are not similar, with a TM‐score of 0.32 and sequence identity of 15.4% (Figure S3B). Taken together, the results suggest that the singleton Pfam domain is possibly a false positive Pfam domain prediction. Thus, while the results are not indicative of a general trend for singleton clusters being false positive Pfam domain predictions, prioritizing singleton Pfam domains with low Pfam prediction confidence scores and high structure prediction confidence scores could be used to identify false positive Pfam domain predictions that can be further used to refine specific Pfam prediction score thresholds. Alternatively, other structure prediction approaches might be considered for Pfam domains not deemed false positives while having low structure prediction confidence scores [43].

### 3.4 Singleton Clusters Facilitate Detection of Structural Outliers in Pfam Families

**FIGURE 4:**
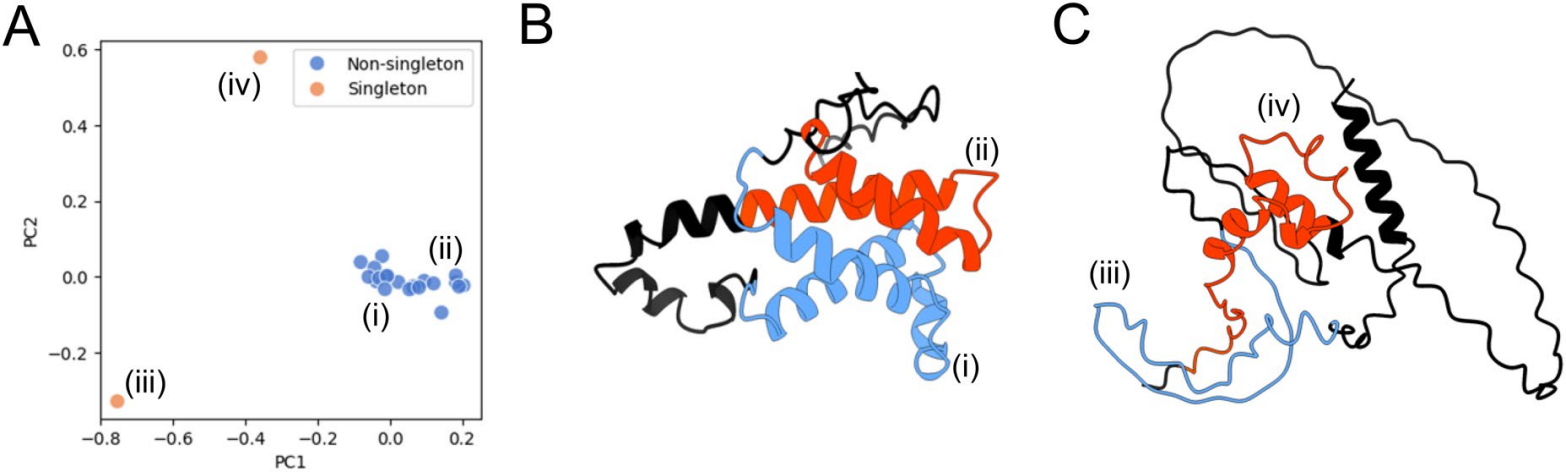
Singleton clusters facilitate detection of structural outliers in Pfam families. (A) PCA plot generated from the TM‐scores between the nodulin PF02451 Pfam family members and agglomerative cluster representatives. Single‐member clusters were labeled as singletons (orange) and multi‐member clusters as non‐singletons (blue). Roman numerals reference specific Pfam domains. (B) The AlphaFold2 structure of A0A0R0GXC2, with both predicted PF02451 Pfam domains highlighted (blue and orange), and referenced with roman numerals (i and ii). (C) The AlphaFold2 structure of P04144, with both predicted PF02451 Pfam domains highlighted (blue and orange, respectively), and referenced with roman numerals (iii and iv, respectively).

The detection of structural outliers in Pfam families provides insights into the structural diversity within these families. As a case study, we selected the nodulin PF02451 Pfam family. Nodulin is a family of plant proteins that have been found to be transcriptionally upregulated in roots during nodulation and are thought to be involved in the development of nodules following rhizobium infection [44, 45]. A PCA plot generated from the TM scores between the cluster representatives and the rest of the Pfam family members shows the distinct PF02451 singleton and non‐singleton clusters (Figure 4A, Table S6). Two non‐singleton Pfam domain structures from the A0A0R0GXC2 nodulin protein are identified as (i) and (ii). In Figure 4B, blue and red colors correspond to the Pfam domain structures for (i) and (ii), respectively, and black is the rest of the protein (Table S6). Both (i) and (ii) structures show an almost overlapping motif of two α‐helices, an expected overlap between predicted structures given that they belong to the same Pfam domain. The orientation of two α‐helices is different, but their relative positioning in the motif forming an X is apparent. Even the helical length of approximately 15 amino acids is within the realistic range of 6–30 [46].

PF02451, however, also contains two singleton clusters from the P04144 nodulin protein, identified as (iii) and (iv) (Table S6). In Figure 4C, blue and red colors correspond to the Pfam domain structures for (iii) and (iv). The (iii) structure is completely made of coil, though the structure looks as if it is about to form a helix (Figure 4C). Similarly, the (iv) structure has some short helical parts with some semblance to the X motif similar to those in structures (i) and (ii) (Figure 4C). At first glance, the structures (i) and (ii) are what one expects to see biologically with respect to the characteristics of secondary structures and protein compactness. The structures (iii) and (iv) are not, however, what one expects from a highly accurate protein structure predictor. Therefore, this case study demonstrates the need for quality control of input sequences and for using several different predicted structures when focusing on a single Pfam family to obtain a range of predicted protein structures to get a sense of what the actual protein structure associated with that specific family may look like in the cell.

### 3.5 Clustering of Pfam Family Structures Can Provide Support for Domain Refinement

**FIGURE 5:**
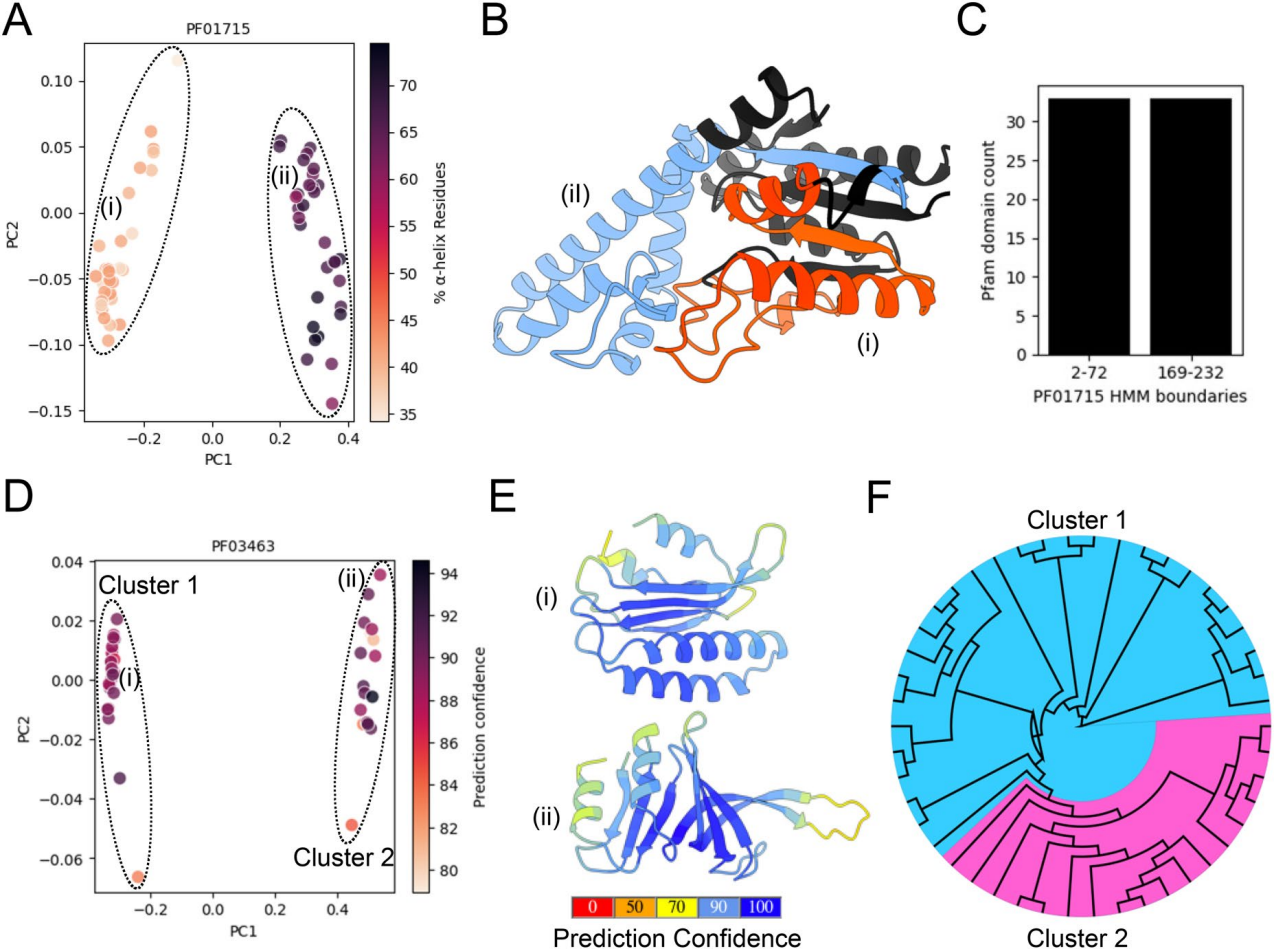
Clustering of Pfam family structures can provide support for domain refinement. (A) PCA plot generated from the TM‐scores between the IPP transferase PF01715 Pfam family members that had two predicted PF01715 domain fragments in the same protein and agglomerative cluster representatives. Individual domain fragments were labeled according to their assigned secondary structure. Roman numerals reference the two predicted Pfam domain fragments of K7MVJ1. (B) The AlphaFold2 structure of K7MVJ1, with both predicted PF01715 Pfam domains highlighted (blue and orange), and referenced with roman numerals (i and ii). (C) Count of the predicted PF01715 Pfam domains based on their predicted overlapping HMM boundaries. HMM boundaries were only predicted for PF01715 Pfam family members with exactly two predicted PF01715 Pfam domains. (D) PCA plot generated from the TM‐scores between the eRF1 PF03463 Pfam family members and agglomerative cluster representatives. The color of individual domains represents prediction confidence scores, quantified as average pLDDT. Roman numerals reference specific Pfam domains. (E) Structure of both cluster representatives, referenced with roman numerals (i for P35614 and ii for I1NAF6) and using the ChimeraX AlphaFold2 color palette for the per‐residue prediction confidence scores (pLDDT). (F) Neighbor‐joining phylogeny tree generated from the PF03463 Pfam family protein domain sequences. The tree was visualized using FigTree and agglomerative clusters were highlighted. Ellipses in PCA plots were added manually to highlight agglomerative clusters.

In addition to the continuous addition of novel Pfam families to the Pfam database, part of the manual curation that goes into the Pfam database involves the refinement of existing Pfam models. In the latest published report from the Pfam database, an emphasis was made on using predicted AlphaFold2 protein structures for curation of new domains and refinement of existing domains [29]. One of the possible outcomes of model refinement is the splitting of existing Pfam families into new models with refined boundaries that better represent conserved protein domains [29]. Clustering of Pfam domain structures is not inherently designed to capture candidates for Pfam domain refinement through domain splitting, as such cases would require the separate prediction of two adjacent Pfam domain fragments with the same Pfam domain HMM profile that are structurally distinct from one another. In an effort to identify these candidates, we parsed all Pfam families with exactly two agglomerative clusters and counted the number of proteins that had overlapping proteins in both clusters (Table S7). The top candidate, PF01715, had the highest number of unique overlapping proteins, 33, that appeared in both clusters of Pfam domains (Table S7). The PF01715 Pfam family is annotated as an isopentenyl pyrophosphate (IPP) transferase enzyme that modifies tRNAs [47]. A PCA plot generated from the TM‐scores between the two cluster representatives and the Pfam domains of the 33 unique overlapping proteins shows the two clusters separated by PC1, with distinct % α‐helix residue content in each cluster (Figure 5A, Table S8). Notably, the PCA plot generated from the TM‐scores between the two cluster representatives and the rest of the Pfam family members suggests a more nuanced association between the clustering and secondary structure assignments when proteins with one predicted Pfam domain are included in the analysis (Figure S2A,B). In Figure 5B, we plotted the AlphaFold2 structure of K7MVJ1, where blue and red colors correspond to the Pfam domain fragment structures for (i) and (ii), both predicted as PF01715 Pfam domains (Figure 5A,B, Table S8). Next, we obtained the HMM profile for the PF01715 Pfam domain and used HMMER [48] to identify the boundaries within the HMM profile itself for the 33 unique proteins that had the domain appear in both clusters (Table S9). The results of this analysis showed that one cluster was consistently associated with HMM boundaries at positions 2–72 and another cluster at positions 169–232 (Figure 5C, Table S9). Although domain refinement through domain splitting requires a more comprehensive analysis of protein sequences, structures, and functional annotations, these results suggest that structure clustering can be a useful approach for identifying and prioritizing Pfam domain families for domain refinement.

Curation and refinement of Pfam families can also be applied to the detection of consistent differences between family members, whether at the sequence or structure level, as evidence to support the splitting of a Pfam family into multiple families [29, 39]. Here, we used the agglomerative clustering results to identify the eRF1 (eukaryotic release factor) PF03463 Pfam family that contains exactly two clusters (Table S10). A PCA plot generated from the TM‐scores between the two cluster representatives and the rest of the Pfam family members shows the two distinct clusters, with most structures having an AlphaFold2 structure prediction confidence score, quantified as average pLDDT, of over 80 (Figure 5D, Table S10). We then plotted the AlphaFold2 structure of the two cluster representative domains, corresponding to the Pfam domain structure of the proteins (i) P35614 and (ii) I1NAF6 (Figure 5E, Table S10). Both structures appear to have different compositions and relative orientations of α‐helices and β‐sheets, with structure (i) having 4 α‐helices and 3 β‐sheets, and structure (ii) having 2 α‐helices and 7 β‐sheets, with most residues having predicted confidence scores, quantified as pLDDT scores, of over 70 (Figure 5E). To test whether the clustering of the Pfam domain structures is reflected in the protein sequences as well, we generated a neighbor‐joining tree from the aligned domain protein sequences, confirming that the Pfam domains cluster together both on the protein sequence level and the structure level (Figure 5D,F). To provide evidence that the proteins in both clusters possess possible distinct functions, we extracted the UniProt names associated with the protein IDs of each Pfam domain, showing that 24 of the 28 Cluster 1 members were named after eRF1, and 13 of the 18 Cluster 2 members were named after PELOTA (Table S10), two protein families with distinct roles in translation termination and mRNA surveillance, respectively [49]. In fact, the results presented here were used during the manuscript peer review process to refine the existing PF03463 Pfam domain by removing the Pelota seed sequences from it and creating a new Pfam domain, PF26356 (Pelota_N).

### 3.6 Analysis of Pfam Domain Structure Clustering Results Can Facilitate the Detection of Uncommon Folds in Structure Predictions

**FIGURE 6:**
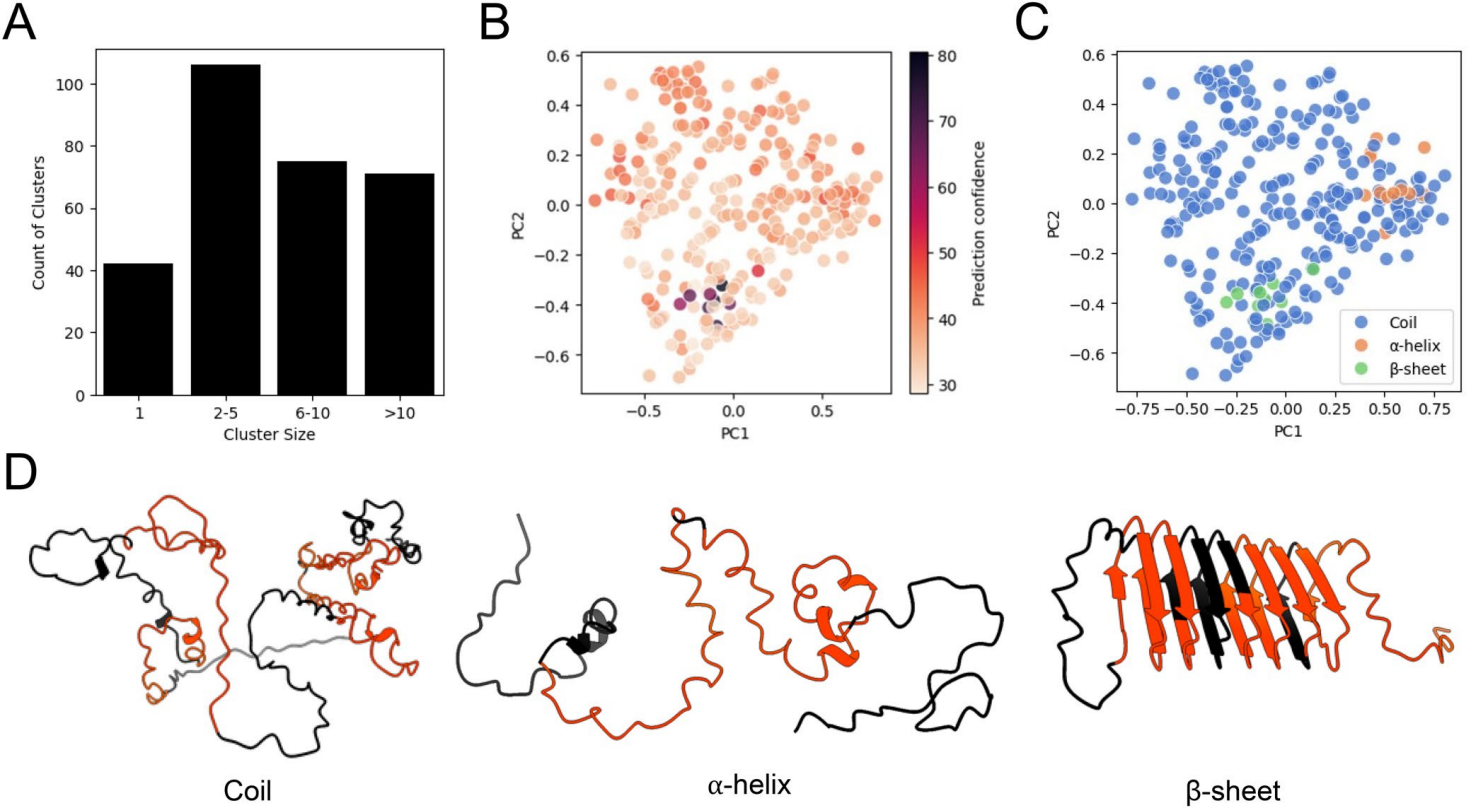
Analysis of Pfam domain structure clustering results can facilitate the detection of uncommon folds in structure predictions. (A) PCA plot generated from the TM‐scores between the keratin‐associated PF13885 Pfam family members and agglomerative cluster representatives. Individual domains were labeled as singleton clusters (orange) or non‐singleton clusters (blue), (B) colored according to prediction confidence scores, quantified as average pLDDT, and (C) according to their assigned secondary structure. (D) The AlphaFold2 structures of M0R935, D4A5R4, and Q9BYR4 were labeled as coil, ⍺‐helix, or β‐sheet, based on the secondary structures assigned to the individual domains in each protein. Individual PF13885 Pfam domains were highlighted in orange across the full structures.

Despite their sequence conservation, some Pfam families can be described as disordered domains, characterized by conserved intrinsic disorder and conserved function [50] making them challenging to structurally align and cluster. The keratin‐associated high sulfur B2 (later termed KAP1) PF13885 Pfam family is thought to form hair fibers in association with hair keratin intermediate filaments [51]. An analysis of the number of agglomerative clusters showed that the PF13885 family contained a total of 294 clusters, 42 singleton clusters, 106 clusters with 2–5 members, 75 clusters with 6–10 members, and 71 clusters with more than 10 members (Figure 6A, Table S11). The PCA plot generated from the TM‐scores between the Pfam family members and agglomerative cluster representatives with the prediction confidence score for the individual Pfam domains, quantified as the average pLDDT, revealed a small cluster containing 8 Pfam domains with confidence scores above 60, while over 96% of Pfam family members had a confidence score below 60 (Figure 6B, Table S11). The PCA plot with the assigned secondary structure categories showed that the high confidence score domains were also assigned a β‐sheet secondary structures, while the α‐helix domains had low confidence scores (Figure 6C, Table S11).

To better understand the structure of the individual domains, we visualized the AlphaFold2 predicted structures of three proteins, M0R935, D4A5R4, and Q9BYR4, with different secondary structure assignments. Labeled as “Coil”, M0R935 contains four PF13885 domains, all assigned to the coil secondary structure (Figure 6D). Labeled as “α‐helix”, D4A5R4 contains two PF13885 domains, one assigned to the α‐helix secondary structure, and one assigned to the coil secondary structure (Figure 6D). Lastly, labeled as “β‐sheet”, Q9BYR4 contains three PF13885 domains, all assigned to a β‐sheet secondary structures (Figure 6D). Importantly, only six of the 120 unique proteins within the PF13885 Pfam family had at least one domain assigned a β‐sheet secondary structure (Table S11).

It is also possible for uncommon folds to arise due to false positive Pfam domain predictions included in the analysis. We compared the Pfam *e*‐value prediction scores against the structure prediction confidence scores for the individual Pfam domains and observed that all but one of the PF13885 Pfam domains assigned to the β‐sheet secondary structure group had low *e*‐value scores, suggesting high Pfam domain prediction confidence (Figure S4A, Table S11). Alternatively, it has been proposed that protein sequence repeats can produce unrealistic solenoid secondary structures by AlphaFold2, but not AlphaFold3 [25]. Because keratin‐associated proteins (KAP) are known to contain multiple dimeric cysteine‐containing pentameric repeats [51], it is possible that their presence plays a role in the observed proteins with β‐sheet secondary structures. Using an alignment of two protein sequences [41], Q3V4B7 (coil) and Q9BYR4 (β‐sheet), we identified a 10 amino acid repeat that appears twice in Q3V4B7 but only once in Q9BYR4 (Figure S4B). After aligning the two protein sequences, we have modified Q3V4B7, labeled Q3V4B7*, to include the second repeat, using Q9BYR4 as a template (Figure S4B). Using the AlphaFold3 webserver [52] to predict the structures of Q9BYR4, Q3V4B7, and Q3V4B7*, and visualizing the results using ChimeraX, we show that by including the second repeat in Q3V4B7*, AlphaFold3 predicted a structure with a relatively high predicted template modeling (pTM) score of 0.43, compared to 0.14 for Q3V4B7 and 0.51 for Q9BYR4, and a distinct β‐sheet secondary structure that more closely resembles Q9BYR4 but is not present in Q3V4B7 (Figure S4C). Thus, we show that by observing the clustering results with overlayed information, such as prediction confidence and assigned secondary structure, we were able to find an uncommon fold in the keratin‐associated PF13885 Pfam family composed of a multiple β‐sheet secondary structures and use the information to provide a tentative explanation for the observed structural diversity.

## 4 Conclusions

Determining the functional roles that proteins play in cells is one of the most complicated questions that only has incomplete answers. Although experimental studies can provide strong evidence for certain protein functions, proteins often have more than a single function, and therefore the issue of experimentally capturing the full range of functional space for a given protein remains a challenge. To fill this knowledge gap, computational methods have been developed for protein function prediction, such as Hidden Markov Models (HMMs) used by the Pfam database, relying on the hypothesis that conserved protein sequences are indicative of conserved protein functions. There are certainly exceptions to this rule, but in general terms, the hypothesis holds and provides a reliable means to predict protein functional spaces. Every predictive method, however, relies on abstractions, parametric models, and a variety of thresholds, and therefore imparts inaccuracies throughout the prediction workflow. This is true for Pfam that uses representative sequences as a basis for functional predictions, as well as powerful machine learning models such as AlphaFold for protein tertiary structure prediction. In this work, we set out to understand the range of imprecision in structural predictions associated with the HMM‐trained Pfam predictions. We have used predicted protein structures across Pfam families and used FoldSeek to cluster these structures to analyze structural diversity. Comparison of the FoldSeek cluster representatives revealed substantial structural similarities between cluster representatives. In order to better capture the structural diversity, we applied agglomerative clustering to merge the FoldSeek clusters to capture diverse structures. We have demonstrated the range of structural diversity within Pfam families and provided case studies to show that structural variability in Pfam families can be used to detect structural outliers and facilitate curation and refinement of Pfam families. The results of our study strongly encourage researchers to survey a wide range of predicted structures for a given Pfam family, so that they can use their scientific judgment and expertise to determine which structures are biologically more meaningful.

## Author Contributions

**Elly Poretsky:** conceptualization, investigation, writing – original draft, writing – review and editing, visualization, methodology. **Carson M. Andorf:** conceptualization, investigation, funding acquisition, writing – review and editing, methodology. **Taner Z. Sen:** conceptualization, investigation, funding acquisition, supervision, project administration, writing – review and editing, methodology.

## Ethics Statement

The authors have nothing to report.

## Consent

The authors have nothing to report.

## Conflicts of Interest

The authors declare no conflicts of interest.

## Supporting information


**Data S1.** Supporting information tables.

**FIGURE S1.** A small number of Pfam families can skew results. (A) Among the 567 Pfam families with at least two clusters remaining follow agglomerative clustering, four Pfam families had more than 1000 singleton clusters, more than the combined number of singleton clusters for the rest 563 Pfam families. (B) Inclusion of the four Pfam families with more than 1000 singleton clusters skews the distribution of prediction confidence scores, average pLDDT, for the singleton clusters.
**FIGURE S2.** Clustering of all the PF01715 Pfam family members. (A) PCA plot generated from the TM‐scores between the IPP transferase PF01715 Pfam family members and agglomerative cluster representatives. Individual domains were labeled according to their (A) assigned cluster representatives and (B) assigned secondary structure.
**FIGURE S3.** A structural outlier singleton cluster is likely a false‐positive Pfam domain prediction. (A) Alignment of the full protein sequences of A0A0N7KDY2 and A0A0N7KQE7, both predicted to have a histidine triad motif (HIT) PF01230 Pfam family domain. The conserved HXHXHXX HIT motif found in A0A0N7KQE7 is bounded by a box. (B) The TM‐align protein structure alignment results for A0A0N7KQE7 (blue) and A0A0N7KDY2 (red) with the reported TM‐score and sequence identity. The protein sequence alignment was conducted using Clustal Omega webserver and the protein structure alignment was conducted using the TM‐align webserver.
**FIGURE S4.** Tentative association between sequence repeats in keratin‐associated proteins (KAP) proteins and the uncommonly observed β‐sheet secondary structure. (A) Scatter plot showing the association between the predicted PF13885 Pfam family domain *E*‐value scores, reported by InterProScan, and structure confidence scores, quantified as average pLDDT. Individual domains were labeled as coil, ⍺‐helix, or β‐sheet, respectively, based on the secondary structures assigned to the individual domains. (B) Alignment of two protein sequences Q3V4B7 (coil) and Q9BYR4 (β‐sheet) with a 10 amino acid repeat highlighted in yellow. Q3V4B7* is a modified version of Q3V4B7 in which Q9BYR4 was used a template to add the second sequence repeat missing from Q3V4B7. (C) The AlphaFold3 predicted structures of Q9BYR4, Q3V4B7, and Q3V4B7*, visualized using the ChimeraX AlphaFold2 color palette for the per‐residue prediction confidence scores (pLDDT).

## Data Availability

A detailed markdown page provides explanations for all analysis steps, code for reproducing results and figures, and links to raw data and results, are available on GitHub at https://github.com/eporetsky/pfamfold. Supporting Information Tables S1–S11 are available for download on the Zenodo data repository at https://zenodo.org/records/15677206.
