# Supplementary material for: Structural Variability of Pfam Domains Based on Alphafold2 Predictions
Source: Proteins. 2025 Jul 22;93(12):2182–92. doi: 10.1002/prot.70021 (PMC12594177; doi:10.1002/prot.70021)
Supplement: Supplementary file 2 — FIGURE S1. A small number of Pfam families can skew results. (A) Among the 567 Pfam families with at least two clusters remaining follow agglomerative clustering, four Pfam families had more than 1000 singleton clusters, more than the combined number of singleton clusters for the rest 563 Pfam families. (B) Inclusion of the four Pfam families with more than 1000 singleton clusters skews the distribution of prediction confidence scores, average pLDDT, for the singleton clusters. FIGURE S2. Clustering of all the PF01715 Pfam family members. (A) PCA plot generated from the TM‐scores between the IPP transferase PF01715 Pfam family members and agglomerative cluster representatives. Individual domains were labeled according to their (A) assigned cluster representatives and (B) assigned secondary structure. FIGURE S3. A structural outlier singleton cluster is likely a false‐positive Pfam domain prediction. (A) Alignment of the full protein sequences of A0A0N7KDY2 and A0A0N7KQE7, both predicted to have a histidine triad motif (HIT) PF01230 Pfam family domain. The conserved HXHXHXX HIT motif found in A0A0N7KQE7 is bounded by a box. (B) The TM‐align protein structure alignment results for A0A0N7KQE7 (blue) and A0A0N7KDY2 (red) with the reported TM‐score and sequence identity. The protein sequence alignment was conducted using Clustal Omega webserver and the protein structure alignment was conducted using the TM‐align webserver. FIGURE S4. Tentative association between sequence repeats in keratin‐associated proteins (KAP) proteins and the uncommonly observed β‐sheet secondary structure. (A) Scatter plot showing the association between the predicted PF13885 Pfam family domain E‐value scores, reported by InterProScan, and structure confidence scores, quantified as average pLDDT. Individual domains were labeled as coil, ⍺‐helix, or β‐sheet, respectively, based on the secondary structures assigned to the individual domains. (B) Alignment of two protein sequences Q3V4B7 (coil) and [file PROT-93-2182-s002.docx]

**Figure S1.** A small number of Pfam families can skew results. **(A)** Among the 567 Pfam families with at least two clusters remaining follow agglomerative clustering, four Pfam families had more than 1,000 singleton clusters, more than the combined number of singletons clusters for the rest 563 Pfam families. **(B)** Inclusion of the four Pfam families with more than 1,000 singleton clusters skews the distribution of prediction confidence scores, average pLDDT, for the singleton clusters.

**Figure S2** Clustering of all the PF01715 Pfam family members. **(A)** PCA plot generated from the TM-scores between the IPP transferase PF01715 Pfam family members and agglomerative cluster representatives. Individual domains were labeled according to their **(A)** assigned cluster representatives and **(B)** assigned secondary structure.

**Figure S3. A structural outlier singleton cluster is likely a false-positive Pfam domain prediction. (A)** Alignment of the full protein sequences of A0A0N7KDY2 and A0A0N7KQE7, both predicted to have a histidine triad motif (HIT) PF01230 Pfam family domain. The conserved HXHXHXX HIT motif found in A0A0N7KQE7 is bounded by a box. (B) The TM-align protein structure alignment results for A0A0N7KQE7 (blue) and A0A0N7KDY2 (red) with the reported TM-score and sequence identity. The protein sequence alignment was conducted using Clustal Omega webserver and the protein structure alignment was conducted using the TM-align webserver.

**Figure S4.** Tentative association between sequence repeats in keratin-associated proteins (KAP) proteins and the uncommonly observed β-sheet secondary structure. **(A)** Scatter plot showing the association between the predicted PF13885 Pfam family domain E-value scores, reported by InterProScan, and structure confidence scores, quantified as average pLDDT. Individual domains were labeled as coil, ⍺-helix, or β-sheet, respectively, based on the secondary structures assigned to the individual domains. **(B)** Alignment of two protein sequences Q3V4B7 (coil) and Q9BYR4 (β-sheet) with a 10 amino acid repeat highlighted in yellow. Q3V4B7* is a modified version of Q3V4B7 in which Q9BYR4 was used a template to add the second sequence repeat missing from Q3V4B7. **(C)** The AlphaFold3 predicted structures of Q9BYR4, Q3V4B7, and Q3V4B7*, visualized using the ChimeraX AlphaFold2 color palette for the per-residue prediction confidence scores (pLDDT).
